# Supplementary material for: MitoHiFi: a python pipeline for mitochondrial genome assembly from PacBio high fidelity reads
Source: BMC Bioinformatics. 2023 Jul 18;24:288. doi: 10.1186/s12859-023-05385-y (PMC10354987; doi:10.1186/s12859-023-05385-y)
Supplement: Supplementary file 2 — Additional file 2. List of species that were assembled by MitoHiFi and that also had previous mitogenomes available on online databases. [file 12859_2023_5385_MOESM2_ESM.pdf]

Additional Table 2: List of species that were assembled by MitoHiFi and that also had previous mitogenomes available on online databases.

**Supplementary Table 2:** Species with previous mitochondrial genome assemblies found on INSC databates. Species in Figure 3A					
Flammulina velutipes					
Acipenser ruthenus					
Agaricus bisporus					
Plebejus argus					
Canis lupus					
Pleurotus ostreatus					
Phragmatobia fuliginosa					
Spilosoma lubricipeda					
Neofelis nebulosa					
Nycticebus coucang					
Pararge aegeria					
Pieris rapae					
Thunnus maccoyii					
Diadumene lineata					
Barbus barbus					
Scomber japonicus					
Orcinus orca					
Cervus elaphus					
Mesoplodon densirostris					
Aporia crataegi					
Eristalis arbustorum					
Syritta pipiens					
Pyrgus malvae					
Xestia c-nigrum					
Thunnus albacares					
Rattus norvegicus					

Melitaea cinxia					
Operophtera brumata					
Pholis gunnellus					
Papilio machaon					
Pieris napi					
Euproctis similis					
Melanostoma mellinum					
Monodelphis domestica					
Calliphora vomitoria					
Mamestra brassicae					
Eristalinus sepulchralis					
Limenitis camilla					
Ocypus olens					
Episyrphus balteatus					
Lumbricus rubellus					
Piscicola geometra					
Coccinella septempunctata					
Sorex araneus					
Accipiter gentilis					
Limnoperna fortunei					
Eristalis tenax					
Lycaena phlaeas					
Platycheirus albimanus					
Plutella xylostella					
Acridotheres tristis					
Pterostichus madidus					
Ischnura elegans					

Eupeodes latifasciatus					
Nebria brevicollis					
Eupeodes corollae					
Tridacna crocea					
Sarcophaga caerulescens					
Tridacna gigas					
Bombus terrestris					
