# Supplementary material for: MitoHiFi: a python pipeline for mitochondrial genome assembly from PacBio high fidelity reads
Source: BMC Bioinformatics. 2023 Jul 18;24:288. doi: 10.1186/s12859-023-05385-y (PMC10354987; doi:10.1186/s12859-023-05385-y)

Additional Figure 1: Dotplots of species mitogenomes, where sequence conservation is represented by a diagonal unique line, and variations, including repeat copies, are shown as deviations from the diagonal. The Y-axis represents mitogenomes assembled by MitoHiFi, while the X-axis represents INSDC mitogenome IDs for the same species assembled using alternative sequencing technologies and by different research groups.

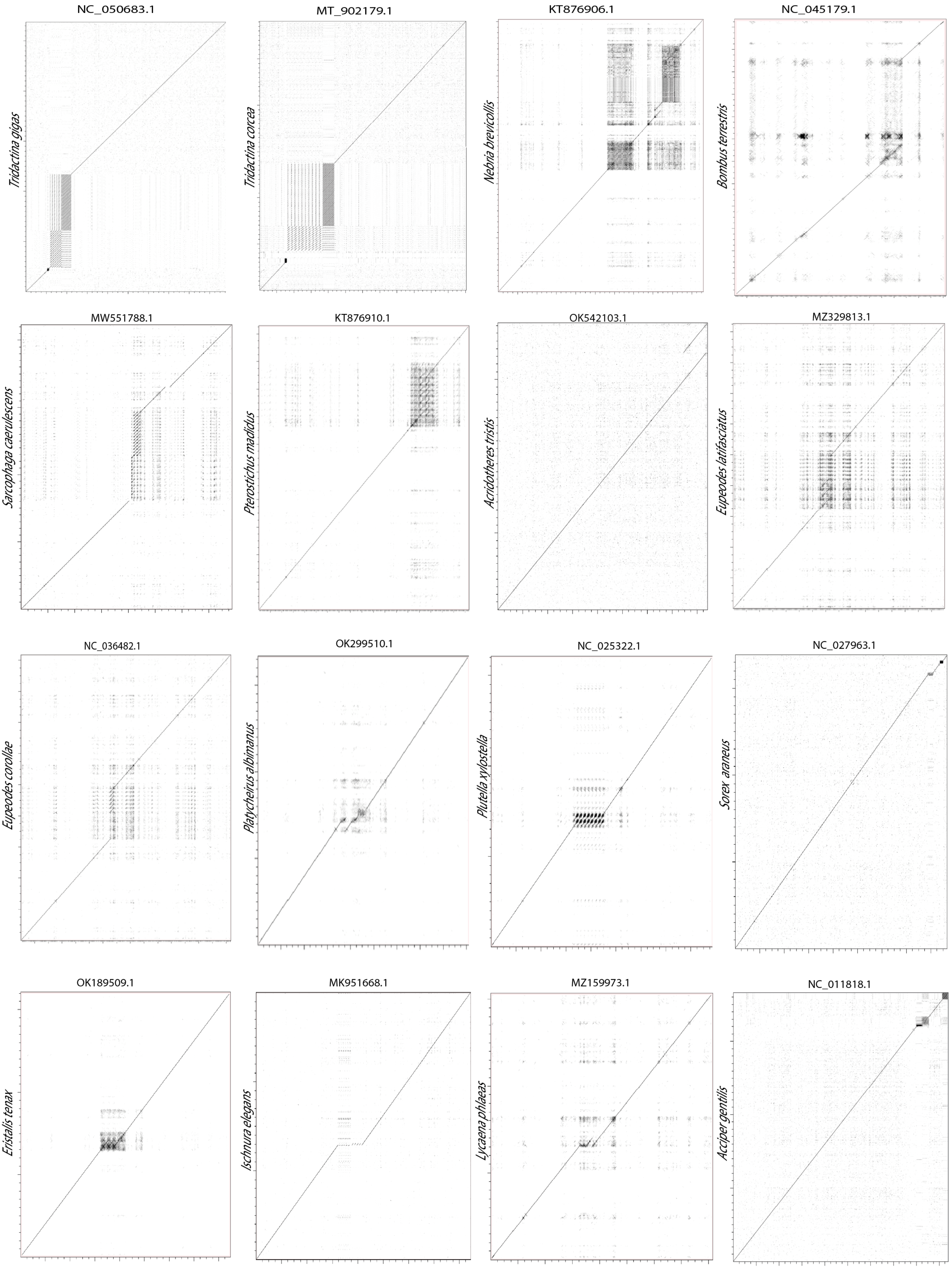

Supplement: Supplementary file 3 — Additional file 3. Figure 1. Dotplots of MitoHiFi mitogenomes (x axis) with their pre-existing mitogenome assemblies (y axis) for each species. [file 12859_2023_5385_MOESM3_ESM.pdf]
