# Supplementary material for: MitoHiFi: a python pipeline for mitochondrial genome assembly from PacBio high fidelity reads
Source: BMC Bioinformatics. 2023 Jul 18;24:288. doi: 10.1186/s12859-023-05385-y (PMC10354987; doi:10.1186/s12859-023-05385-y)
Supplement: Supplementary file 4 — Additional file 4. Figure 2. Screenshots of IGV plots of reads mapped back to Andrea bucephala mitogenome before and after NUMTs reads were removed. [file 12859_2023_5385_MOESM4_ESM.pdf]

Additional Figure 2: Screenshots of IGV plots of reads mapped back to Andrea bucephala mitogenome before and after NUMTs reads were removed.

A

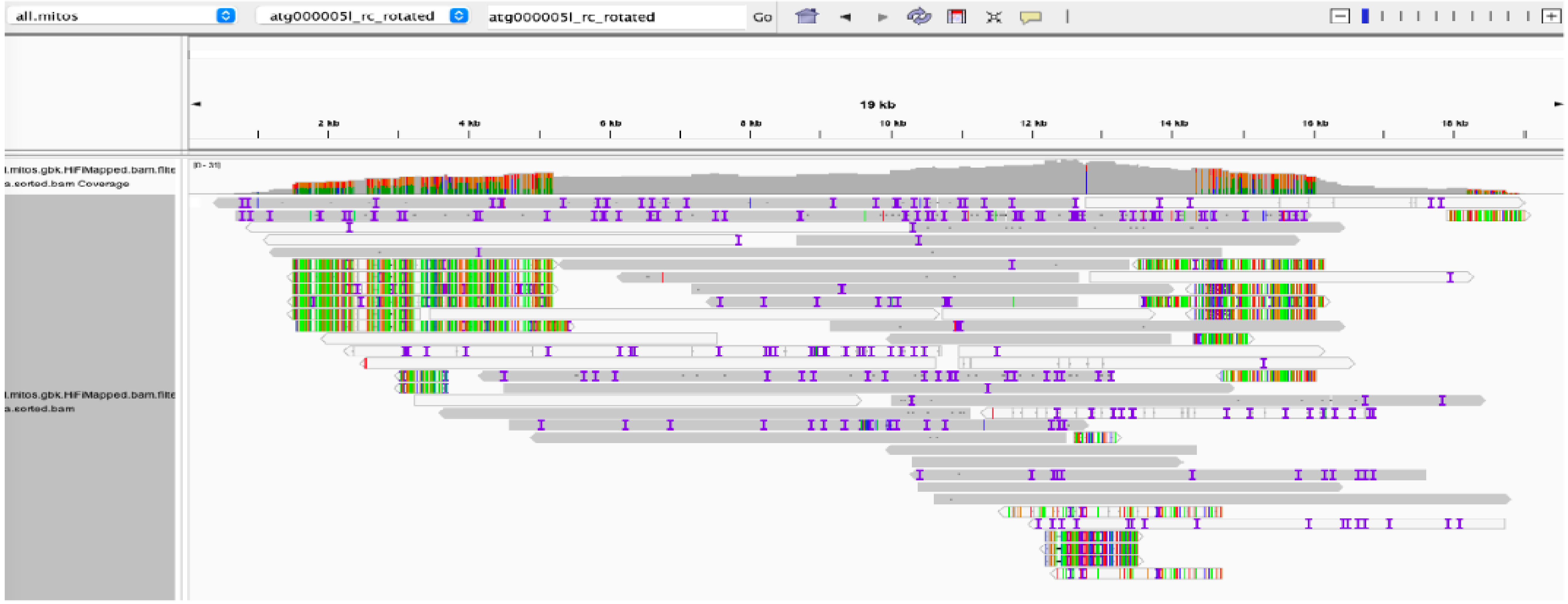

B

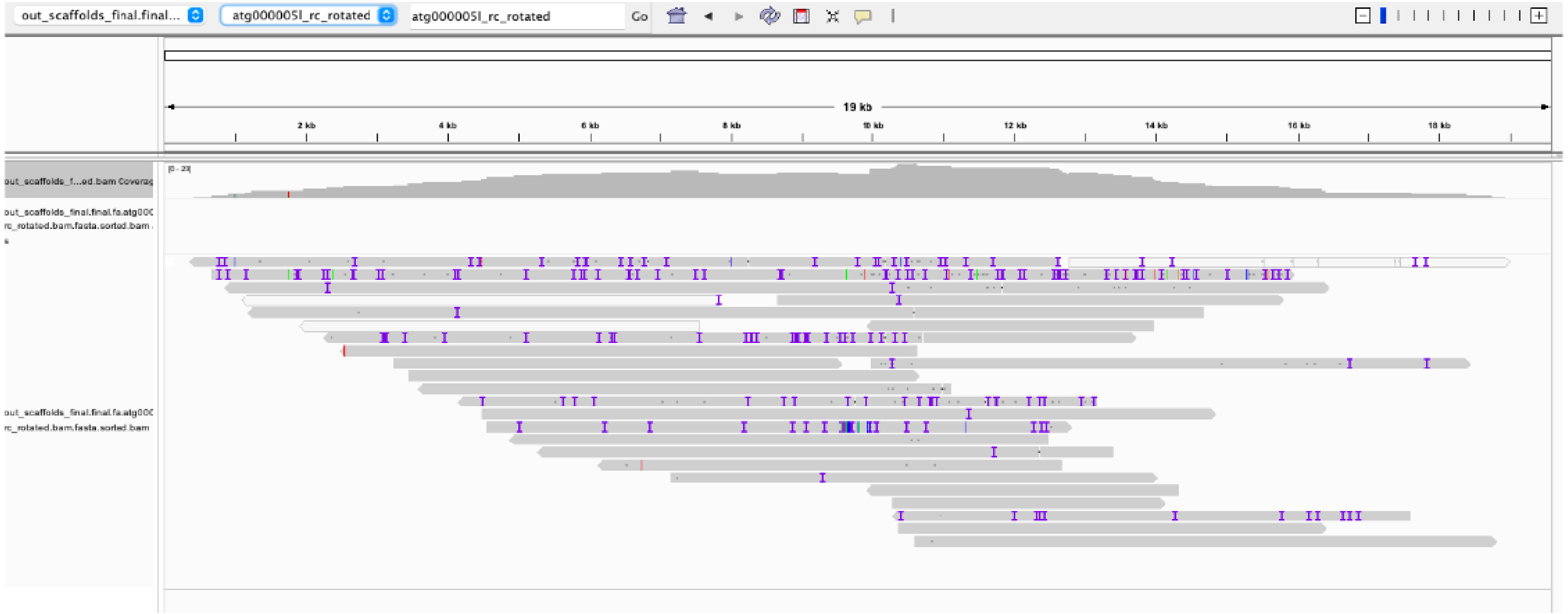

Supplementary Figure 2: Panel **A** shows an IGV plot of all MitoHiFi filtered reads mapped back to the final_mitogenome.fasta of *Andrena bucephala.* In Panel **B**, when reads are mapped to the final_mitogenome.fasta together with the nuclear genome, NUMTs reads are attracted to map at their nuclear locations and the variation track is gone.
